# Differences in MS clinical and epidemiological characteristics between Ashkenazi and non-Ashkenazi Jewish patients in Israel: a retrospective single center study

**DOI:** 10.1038/s41598-022-08565-7

**Published:** 2022-03-16

**Authors:** Arnon Karni, Gil Ben Noon, Tamara Shiner, Ifat Vigiser, Hadar Kolb, Keren Regev

**Affiliations:** 1 Neuroimmunology and Multiple Sclerosis Unit, Department of Neurology, Tel Aviv Sourasky Medical Center, Tel Aviv, Israel; 2 Sackler Faculty of Medicine, Tel Aviv University, Tel Aviv, Israel; 3 Sagol School of Neuroscience Tel Aviv University, Tel Aviv, Israel

**Keywords:** Multiple sclerosis, Epidemiology

## Abstract

The prevalence and severity of Multiple Sclerosis (MS) varies across different ethnicities, with a tendency to a more severe phenotype in non-Caucasian populations.  Our objective was to evaluate the differences in disease phenotype between Ashkenazi Jewish and Non-Ashkenazi Jewish patients in Israel. We conducted a single center retrospective cohort study in which subjects were assigned to Ashkenazi or Non-Ashkenazi groups according to self-reported ancestry and disease severity was assessed using the expanded disability status (EDSS), MS severity score (MSSS), progression index (PI) and MRI metrics. 330 Ashkenazi Jewish (AJ) and 207 Non-Ashkenazi Jewish patients (Non-AJ) were included. Non-AJ had a younger age of disease onset (32.7 years vs. 35.7 years, *p* = 0.05), with a lower proportion of females (62.3% vs. 73.3%, *p* = 0.01). These differences were maintained within the subgroup of Israeli native patients. Ethnicity was a significant predictor of MSSS (β = 0.601, *p* = 0.003), with a higher estimate than that of other epidemiological factors. To conclude, Non-AJ patients had an earlier age of onset and a more disabling disease as well as having a more balanced female to male ratio compared to AJ patients. These findings demonstrate variability of disease phenotype within Caucasian patient's dependent on their ethnicity despite equivalent access to healthcare services.

## Introduction

Multiple sclerosis (MS) is an autoimmune disease characterized by demyelination and subsequent neurodegeneration of the central nervous system (CNS) and is a leading cause of neurological disability in the young^1^. Disease phenotype is highly diverse and is influenced by various genetic and environmental variables^2,3^. The risk of developing MS is different across geographic areas with higher prevalence in North America and Europe compared with Africa, Asia and South America, and with a latitude gradient^4^. Ethnicity modulates the risk of developing MS with lower prevalence recorded in African and Asian minorities living in the United States and Europe, compared to Caucasians^5–7^. The rate of MS among immigrants is found to be more affected by the rate of the disease in the country they migrated to than the rate in their country of origin, with greater susceptibility in childhood^8^ suggesting that environmental factors act early in life in genetically susceptible individuals and drive disease evolution. The age-dependent effect of migration on MS frequency has also been described in Afro-Asian immigrants in Israel^9^.

In the US, African Americans with MS were found to develop disability more frequently and more rapidly than Caucasian Americans^10–12^. Furthermore, it was demonstrated that African Americans die from the disease at an earlier age than Non-Hispanic whites^13^. Other observations of worse MS outcomes in non-white ethnic minorities were described in Europe^14–16^, Iran^17^ and Canada^18^.

Jewish people in Israel are considered to be all of Caucasian decent, although great variation in ethnicity is found even among this group with a differentiation between Ashkenazi Jews (Immigrants from Center and East Europe and North America) and Non-Ashkenazi Jews (immigrants from the Middle East and North Africa). The prevalence of MS in Israel and associated ethnic factors has been previously evaluated in Ashkenazi Jews and non-Ashkenazi Jews and their descendants born in Israel^19,20^, and in the non-Jewish population in Israel, composed of Christian and Muslim Arabs, Druze, and Bedouins^21^. Greater prevalence was recorded among Ashkenazi Jews compared to non-Ashkenazi Jews, and amongst the latter compared with Arabs in Israel.

In 1999 Kwon et al. demonstrated different HLA profiles in Ashkenazi Jews with MS compared to non-Ashkenazi Jews^22^, an observation which could potentially explain a difference in clinical phenotype across these groups. The assumption that an association between ethnicity and clinical course in the Jewish population in Israel was tested in 1964 by Alter et al. but no differences in phenotype was demonstrated in the two ethnic groups other than an earlier age of onset in Non-Ashkenazi Jews^23^. The study included 269 cases of which 25 were born in Israel. Since then, the Israeli population has changed demographically with new waves of immigration and generations of native citizens. Additionally, new tools and clinical measures of MS activity have been developed and the use of Magnetic Resonance Imaging (MRI) became significant in disease evaluation^24^.

We hypothesized that non-Ashkenazi MS patients would demonstrate a more aggressive disease phenotype, with greater neurological disability over time. Understanding MS disease course in specific populations can provide insight into genetic and environmental factors that affect MS disease pathogenesis and assists in precision medicine approaches.

## Methods

A retrospective study was conducted in the Neuroimmunology and Multiple Sclerosis Unit at the Tel Aviv Sourasky Medical Center. The Institutional Review Board approved the study (No. 0597-17-TLV). Data was collected from medical records of all patients who visited the clinic between January 2018 and August 2020 and included demographic details and clinical evaluation including Expanded Disability Status Score (EDSS) assessment^25^. Data regarding MRI disease burden and activity was collected from neuroradiological reports, using scans preformed closest to the time of EDSS assessment. Missing data regarding demographic background was completed by direct questioning. Only patients with a confirmed diagnosis of MS according to 2017 McDonald's criteria, disease duration of at least 1 year and a known homogenous ethnic affiliation of (i.e., only Ashkenazi or only Non-Ashkenazi) were included.

We evaluated disease progression using the MS severity score (MSSS), a validated measure derived from the EDSS and disease duration^26^. The MSSS is a powerful and validated tool for the estimation of disease severity over time based on single assessment data, and it has shown stability over time when computed for patients with disease duration of at least 1 year. Additionally, the Progression Index (PI) was computed, reflecting the ratio between EDSS and disease duration. Other outcomes accounted for in the comparison are spinal cord and infra-tentorial involvement in MRI, proportion of patients with an EDSS of 6 or higher, MS course (relapsing remitting, secondary progressive and primary progressive) and presence of oligoclonal bands (OCB) in the CSF. Treatment strategy was added as a variable by dividing the patients to four levels of therapy; untreated, treated with a platform agent (i.e., Glatiramer Acetate, Interferon β, Dimethyl Fumarate and Teriflunomide), induction therapy with high efficacy agent (i.e., Fingolimod, Alemtuzumab, Cladribine, Natalizumab, Ocrelizumab and Siponimod), and escalation therapy (e.g., transition from a platform agent to high efficacy agent).

### Statistical analysis

Statistical analysis was performed using R-studio 4.0 with the *ms.sev* package utilized to compute MSSS scores. Comparison between ethnic groups was performed using Chi-square test for categorial variables, and student's *t* or Mann Whitney U tests for continuous variables according to normality assessed by Shapiro test.

ANCOVA tests were carried out to control MSSS results for age at disease onset and age at assessment, once confirming that the appropriate assumptions were met.

Differences in clinical parameters and scores between ethnic groups were evaluated with stratification to sex, MS subtype and immigration status (immigrant versus Israeli native).

Ethnic affiliation was evaluated as a predictor of MSSS in a univariate linear regression model including other variables with effect on disease severity. ANOVA test was used to assess wither adding ethnicity to the model significantly improved fit.

### Ethics approval and consent to participate

The study was conducted in accordance with the 1964 Declaration of Helsinki and its later amendments. The study was approved by the Tel Aviv Sourasky Medical Center Institutional Review Board (Helsinki Committee, No. 0597-17-TLV). The need for consent was waived by the Tel Aviv Sourasky Medical Center Institutional Review Board in due to the retrospective nature of the study.

## Results

The registry of the MS clinic in Tel Aviv medical center yielded 730 updated patient profiles, of which 537 met the inclusion criteria. 330 patients identified themselves as Ashkenazi Jews (AJ) and 207 patients identified themselves as Non-Ashkenazi Jews (Non-AJ), according to their ancestry (Table 1). Non-AJ patients were younger on average at the point of data collection (43.0 ± 14.1 years vs. 49.4 ± 14.5 years, *p* value < 0.001), and included fewer females (62.3% vs. 73.3%, *p* value = 0.010) compared with AJ patients. Fewer patients in the Non-AJ group were immigrants (14.0% vs. 54.8%, *p* value < 0.001) and the median age at immigration in this group was lower (12.5 years vs. 21.5 years, *p* value = 0.043) compared with Ashkenazi patients. No significant differences were found in comorbidities between the groups. Family history of MS was more frequent among Non-AJ patients (10.1% vs. 3.94%, *p* value = 0.007), while differences in family history of other neurologic or autoimmune comorbidities did not reach significance. Smoking habits did not differ between groups.

**Table 1:** Characteristics of study population.

	Ashkenazi, * N* = 330	Non-Ashkenazi, * N* = 207	*p* value
Age (years); mean (sd)	49.4 (14.5)	43.0 (14.1)	** < 0.001**
Female; *n* (%)	242 (73.3%)	129 (62.3%)	**0.010**
Immigrant; *n* (%)	181 (54.8%)	29 (14.0%)	** < 0.001**
Age at immigration median [1stQ, 3rdQ]	21.5 [11.0; 29.0]	12.5 [6.75; 23.5]	**0.043**
**Region of birth; *n* (%)**			NA
Africa	0 (0.00%)	6 (2.90%)	
Central Asia	2 (0.61%)	0 (0.00%)	
East-Central Europe	25 (7.58%)	0 (0.00%)	
Israel	149 (45.2%)	178 (86.0%)	
North America	20 (6.06%)	1 (0.48%)	
North Europe	2 (0.61%)	0 (0.00%)	
South America	2 (0.61%)	1 (0.48%)	
Former USSR	118 (35.8%)	7 (3.38%)	
West Asia	0 (0.00%)	9 (4.35%)	
West Europe	12 (3.64%)	5 (2.42%)	
Smoking; *n* (%)	48 (14.5%)	44 (21.3%)	0.059
Systemic comorbidities; *n* (%)	56 (17.0%)	30 (14.5%)	0.522
Neurological comorbidities; *n* (%)	8 (2.42%)	8 (3.86%)	0.487
Oher autoimmune comorbidities; *n* (%)	43 (13.0%)	16 (7.73%)	0.077
Family history of MS; *n* (%)	13 (3.94%)	21 (10.1%)	**0.007**
Family history of neurological conditions; *n* (%)	14 (4.24%)	12 (5.80%)	0.542
Family history of other autoimmune conditions; *n* (%)	20 (6.06%)	20 (9.66%)	0.198

Comparison of demographic characteristics in Ashkenazi patients vs. non-Ashkenazi patients. Variables that were normally distributed according to Shapiro test (*p* value < 0.05) are presented in the format of mean (SD) and compared using Student T-test. Non-normal variables are presented by median [1st quartile, 3rd quartile], and compared using Mann-Whitney *U *test. Categorial variables are compared using Chi-squared test and presented with count (%).

*p* values in bold denote statistical significance (*p* < 0.05).

*USSR* Union of Soviet Socialist Republics, *MS* multiple sclerosis.

### Disease phenotype

Analysis of differences in disease phenotype is presented in Table 2. Non-AJ patients, on average, were younger at disease onset (32.7 ± 11.9 years vs. 35.7 ± 12.1 years, *p* value = 0.005) and had shorter median disease duration at the time of EDSS evaluation (9.0 years vs. 12.0 years, *p* value < 0.001) compared with AJ patients (Table 2). The mean MSSS was significantly higher in Non-AJ patients when controlling for age at EDSS evaluation (3.50 ± 0.19 vs. 2.78 ± 0.15, *p* value = 0.005) and age at onset (3.41 ± 0.19 vs. 2.84 ± 0.15, *p* value = 0.023) using ANCOVA. The groups did not differ in PI, proportion of patients with EDSS 6 or above, MRI metrics, treatment strategy, MS subtype and OCB profile.

**Table 2:** Disease phenotype.

	Ashkenazi (*N* = 330)	Non-Ashkenazi (* N* = 207)	*p* value
Age at onset; mean (SD)	35.7 (12.1)	32.7 (11.9)	**0.005**
Disease duration (years); median [1stQ, 3rdQ]	12.0 [5.00;20.0]	9.00 [4.00;15.0]	** < 0.001**
EDSS; median [1stQ, 3rdQ]	2.00 [1.00;4.00]	2.00 [0.00;4.75]	0.917
Global MSSS; mean (SD)	2.91 (2.87)	3.29 (3.00)	0.149
Global MSSS; mean (SE), covariate: age at EDSS	2.78 (0.15)	3.50 (0.19)	0.005*
Global MSSS; mean (SE, covariate: age at onset	2.84 (0.15)	3.41 (0.19)	0.023**
PI; median [1stQ, 3rdQ]	0.17 [0.04;0.36]	0.21 [0.00;0.50]	0.188
EDSS 6.0 and above; n (%)	63 (19.1%)	43 (20.8%)	0.715
Spinal cord involvement in MRI; n (%)	188 (57.0%)	115 (55.6%)	0.990
Posterior Fossa involvement in MRI; n (%)	152 (46.1%)	95 (45.9%)	1.000
**Treatment strategy; n (%)**			0.844
Untreated	31 (9.42%)	23 (11.1%)	
Platform therapy	174 (52.9%)	102 (49.3%)	
High efficacy	42 (12.8%)	27 (13.0%)	
Escalation therapy	82 (24.9%)	55 (26.6%)	
**MS subtype; n (%)**			0.368
PPMS	31 (9.39%)	13 (6.28%)	
RRMS	258 (78.2%)	171 (82.6%)	
SPMS	41(12.4%)	23(11.1%)	
Oligoclonal bands in CSF; n (%)	118(76.6%)	102(81.0%)	0.464

This table presents the comparison of disease outcomes by ethnic groups. Variables that were normally distributed according to Shapiro test (*p* value < 0.05) are presented in the format of mean (SD) and compared using Student T-test. The MSSS was compared using student T-test as accepted for continuous scales in large samples. Non-normal variables are presented by median [1st quartile, 3rd quartile], and compared using Mann-Whitney *U *test. Categorial variables are compared using Chi-squared test and presented with count (%). *p* values in bold denote statistical significance (p < 0.05). As post-hoc analysis estimated marginal means (standard error) are presented for MSSS adjusted to covariates using ANCOVA. *p *values marked by one asterisk (*) are controlled for age at EDSS, while those marked with 2 asterisks (**) are controlled for age at onset using ANCOVA.

*EDSS* Expended Disability Status Score, *MSSS* Multiple Sclerosis Severity Score, *PI* Progression Index, *MRI* magnetic resonance imaging, *PPMS* primary progressive multiple sclerosis, *RRMS* relapsing remitting multiple sclerosis, *SPMS* secondary progressive multiple sclerosis, *CSF* cerebrospinal fluid.

### Phenotype by disease subtypes

Analysis of differences in disease phenotype by disease subtype is presented in Table 3. When patients with RRMS were analyzed separately trends regarding age at EDSS evaluation, age at onset and disease duration were similar to previously described for the general cohort. The mean MSSS was higher among Non-AJ patients as compare to AJ patients (2.47 ± 2.52 vs. 1.90 ± 2.11, *p* value = 0.014), and was consistently significant when controlling for age at EDSS and age at disease onset using ANCOVA.

**Table 3:** Stratification by disease subtype.

	PPMS					RRMS				SPMS		
	Ashkenazi		Non-Ashkenazi		*p* value	Ashkenazi	Non-Ashkenazi		*p* value	Ashkenazi	Non-Ashkenazi	*p* value
	N = 31		N = 13			N = 258	N = 171			N = 41	N = 23	
Age (years); mean (SD)	60.4 (11.7)		54.4 (12.3)		0.150	46.7 (14.1)	41.0 (13.7)		** < 0.001**	58.2 (10.8)	51.8 (11.9)	**0.040**
Female; n (%)	20 (64.5%)		3 (23.1%)		**0.029**	192 (74.4%)	112 (65.5%)		0.060	30 (73.2%)	14 (60.9%)	0.461
Age at onset; median [1stQ, 3rdQ]/RRMS: mean (SD)	49.0 [36.5; 54.5]		46.0 [43.0; 50.0]		0.806	34.3 (11.2)	31.7 (11.4)		**0.017**	35.0 [29.0; 45.0]	33.0 [21.5; 43.0]	0.313
Disease duration (years); median [1stQ, 3rdQ]	11.0 [6.50; 20.5]		7.00 [5.00; 10.0]		0.073	11.0 [5.00; 17.0]	7.00 [3.00; 13.0]		**0.001**	22.0 [16.0; 27.0]	19.0 [11.5; 24.5]	0.167
EDSS; median [1stQ, 3rdQ]	6.50 [4.25; 6.75]		6.00 [5.50; 7.00]		0.602	1.00 [0.00; 2.00]	1.00 [0.00; 3.00]		0.536	6.50 [6.00; 7.00]	6.50 [5.75; 7.00]	0.983
Global MSSS; median [1stQ, 3rdQ]/RRMS: mean (SD)	7.54 [5.09; 8.69]		8.24 [7.65; 8.83]		0.116	1.90 (2.11)	2.47 (2.52)		**0.014** 0.012* 0.007**	6.63 [5.02; 8.15]	6.57 [4.61; 8.16]	0.679 0.903*
PI; median [1stQ, 3rdQ]	0.54 [0.29; 0.90]		1.00 [0.75; 1.10]		**0.046**	0.11 [0.00; 0.25]	0.15 [0.00; 0.39]		0.165	0.29 [0.21; 0.36]	0.35 [0.24; 0.55]	0.130
EDSS 6.0 and above; n (%)	18 (58.1%)		9 (69.2%)		0.723	13 (5.04%)	17 (9.94%)		0.079	32 (78.0%)	17 (73.9%)	0.946
Spinal cord involvement in MRI; n (%)	24 (77.4%)		10 (76.9%)		1.000	139 (53.9%)	92 (53.8%)		0.901	25 (61.0%)	13 (56.5%)	0.934
Posterior Fossa involvement in MRI; n (%)	14 (45.2%)		5 (38.5%)		0.940	116 (45.0%)	75 (43.9%)		0.900	22 (53.7%)	15 (65.2%)	0.526
Treatment strategy; n (%)					0.672				0.231			0.461
Untreated	3 (9.68%)		1 (7.69%)			27 (10.5%)	22 (12.9%)			1 (2.44%)	0 (0.00%)	
Platform therapy	1 (3.23%)		1 (7.69%)			160 (62.3%)	96 (56.1%)			13 (31.7%)	5 (21.7%)	
High efficacy	19 (61.3%)		6 (46.2%)			17 (6.61%)	20 (11.7%)			6 (14.6%)	1 (4.35%)	
Escalation therapy	8 (25.8%)		5 (38.5%)			53 (20.6%)	33 (19.3%)			21 (51.2%)	17 (73.9%)	

This table presents differences in disease phenotype between groups with stratification to disease subtype. Variables that were normally distributed according to Shapiro test (*p* value < 0.05) are presented in the format of mean (SD) and compared using Student T-test. The MSSS in the RRMS subgroup was compared using student T-test as accepted for continuous scales in large samples. Non-normal variables are presented by median [1st quartile, 3rd quartile], and compared using Mann-Whitney *U *test. Categorial variables are compared using Chi-squared test and presented with count (%). *p* values in bold denote statistical significance (p < 0.05). *p *values marked by one asterisk (*) are controlled for age at EDSS, while those marked with 2 asterisks (**) are controlled for age at onset using ANCOVA.

Normally distributed variables are compared using student T test and are presented with mean (SD), while non-normal variables are compared using Mann Whitney U test and are presented with median [1st quartile, 3rd quartile].

*PPMS* primary progressive multiple sclerosis, *RRMS* relapsing remitting multiple sclerosis, *SPMS* secondary progressive multiple sclerosis, *EDSS* Expended Disability Status Score; *MSSS* Multiple Sclerosis Severity Score, *PI* Progression Index, *MRI* magnetic resonance imaging.

*Controlled for age at EDSS using ANCOVA.

**Controlled for age at onset using ANCOVA.

No differences in disease phenotype were found between patients with SPMS. Non-AJ patients with PPMS had a higher median PI (1.00 vs. 0.54 , *p* value = 0.046).

### Phenotype by sex

Analysis of differences in disease phenotype by sex is presented in Table 4. Mean MSSS was significantly higher amongst Non-AJ female patients as compared to AJ female patients when controlled for age at EDSS (2.93 ± 2.81 vs. 2.78 ± 2.91, *p* value = 0.043), but not when controlled for age at disease onset. Among males no differences in phenotype were found.

**Table 4:** Disease phenotype by sex.

	Female			Male		
	Ashkenazi	Non-Ashkenazi	*p* value	Ashkenazi	Non-Ashkenazi	*p* value
	N = 242	N = 129		N = 88	N = 78	
Age (years); mean (SD)	50.0 (15.1)	43.2 (14.6)	** < 0.001**	47.7 (12.6)	42.8 (13.4)	**0.016**
Age at onset; mean (SD)	36.3 (12.5)	32.8 (12.4)	**0.010**	34.0 (10.8)	32.5 (11.0)	0.396
Disease Duration (years); median [1stQ, 3rdQ]	12.0 [5.00; 20.0]	9.00 [3.00; 15.0]	**0.002**	11.5 [5.00; 19.0]	8.00 [4.25; 14.8]	**0.027**
EDSS; median [1stQ, 3rdQ]	1.75 [0.00; 4.00]	1.50 [0.00; 4.00]	0.678	2.00 [1.00; 5.50]	2.25 [1.00;6 .00]	0.988
Global MSSS; mean (SD)/median [1stQ, 3rdQ]	2.78 (2.91)	2.93 (2.81)	0.637 0.043* 0.108**	2.43 [0.85; 5.36]	2.62 [0.90; 6.82]	0.339 0.164*
PI; median [1stQ, 3rdQ]	0.14 [0.00; 0.33]	0.17 [0.00; 0.43]	0.528	0.20 [0.10; 0.38]	0.26 [0.09; 0.61]	0.242
EDSS 6.0 and above; n (%)	196 (81.0%)	108 (83.7%)	0.611	71 (80.7%)	56 (71.8%)	0.244
Spinal cord involvement in MRI; n (%)	137 (56.6%)	69 (53.5%)	0.641	51 (58.0%)	48 (61.5%)	0.756
Posterior Fossa involvement in MRI; n (%)	102 (42.1%)	56 (43.4%)	0.901	50 (56.8%)	39 (50.0%)	0.470
**Treatment strategy; n (%)**			0.705			0.169
Untreated	23 (9.54%)	13 (10.1%)		8 (9.09%)	10 (12.8%)	
Platform therapy	132 (54.8%)	77 (59.7%)		42 (47.7%)	25 (32.1%)	
High efficacy	33 (13.7%)	13 (10.1%)		9 (10.2%)	14 (17.9%)	
Escalation therapy	53 (22.0%)	26 (20.2%)		29 (33.0%)	29 (37.2%)	
**MS subtype; n (%)**			0.063			0.982
PPMS	20 (8.26%)	3 (2.33%)		11 (12.5%)	10 (12.8%)	
RRMS	192 (79.3%)	112 (86.8%)		66 (75.0%)	59 (75.6%)	
SPMS	30 (12.4%)	14 (10.9%)		11 (12.5%)	9 (11.5%)	
Oligoclonal bands in CSF; n (%)	85 (78.0%)	63 (80.8%)	0.779	33 (73.3%)	39 (81.2%)	0.506

This table presents differences in disease phenotype between groups with stratification to sex. Variables that were normally distributed according to Shapiro test (*p* value < 0.05) are presented in the format of mean (SD) and compared using Student T-test. The MSSS in females was compared using student T-test as accepted for continuous scales in large samples. Non-normal variables are presented by median [1st quartile, 3rd quartile], and compared using Mann-Whitney *U *test. Categorial variables are compared using Chi-squared test and presented with count (%). *p* values in bold denote statistical significance (p < 0.05). *p *values marked by one asterisk (*) are controlled for age at EDSS, while those marked with 2 asterisks (**) are controlled for age at onset using ANCOVA.

*EDSS* Expended Disability Status Score, *MSSS* Multiple Sclerosis Severity Score, *PI* Progression Index, *MRI* magnetic resonance imaging, *PPMS* primary progressive multiple sclerosis, *RRMS* relapsing remitting multiple sclerosis, *SPMS* secondary progressive multiple sclerosis, *CSF* cerebrospinal fluid.

### Phenotype by sex and disease subtype

When considering both strata by disease subtype and by sex, patients with SPMS and PPMS form small samples that limit the analysis, therefore this section centered on patients with RRMS (Supplementary table 1).

In female patients with RRMS mean MSSS was higher in Non-AJ patients (2.40 ± 2.55 vs. 1.76 ± 2.12, *p* value = 0.026), and remained so when controlled for age at EDSS (*p* value = 0.011) and age at onset using ANCOVA (*p* value = 0.008).

Differences in treatment strategy were noted between AJ and Non-AJ male patients with RRMS; more Non-AJ patients were untreated at the time of data collection (15.3% vs. 9.1%, *p* value = 0.026), but amongst those receiving DMT, Non-AJ patients were more often prescribed with high efficacy agents at disease onset (16.9% vs 3.0%, *p* value = 0.026), and less often with platform therapies (42.4% vs. 60.6%, *p* value = 0.026).

### Phenotype in Israeli natives and in immigrants

Among Israeli natives, MSSS was higher (3.25 ± 3.03 vs. 2.76 ± 2.84, *p* value = 0.136) in native Non-AJ patients who were born in Israel, with significance when controlled for age at EDSS evaluation (*p* value = 0.009) and age at onset (*p* value = 0.034) using ANCOVA (Supplementary table 2).

No differences were found between AJ and Non-AJ patients who immigrated to Israel; however, the analysis was likely limited by imbalance in groups’ size.

### Predictability of MSSS by ethnic affiliation in a multivariate linear regression model

In order to evaluate the contribution of ethnicity to the prediction of disease severity, MSSS was regressed as an outcome in two models; model 1 was fitted based on ethnicity, MS subtype, sex, age at onset,smoking and exposure to high-efficacy therapy, in a second model (model 2) ethnicity was not included for the benefit of comparing the models. To fit the models, we added terms for variables that affect disease severity. MS subtype naturally indicates disease progression, and indeed patients with SPMS and PPMS had a higher mean MSSS than those with RRMS (6.60 and 7.97 respectively vs. 1.28). Male sex is a known risk factor for malignant MS^27^, in accordance with this, males in our cohort had a higher mean MSSS than females (3.57 vs. 2.83, *p* = 0.008). Age at onset and age at EDSS evaluation were positively correlated with MSSS (r = 0.258 and r = 0.249 respectively, *p* < 0.001), but were also correlated to one another and therefore the model was fitted with age at onset alone, an established factor in disease severity^27^. Smokers had a higher median MSSS than non-smokers (2.44 vs. 1.77, *p* = 0.040), as reported in the literature^28^. Although no changes were found between groups regarding treatment strategy, treatment with high efficacy drugs is known to affect prognosis^29^. Therefore, treatment was added to the model factored to two levels: patients who never received a high efficacy drug, and patients who received high efficacy drugs either as first line treatment or as an escalation strategy. Immigrants were not found to have a higher MSSS thus immigration status was not included in the model. Due to missing data regarding the presence of OCB in the CSF, and since no significant differences were found in this variable between the groups, this factor was not included in the model, in order to preserve adequate sample size for the analysis.

**Figure 1:**
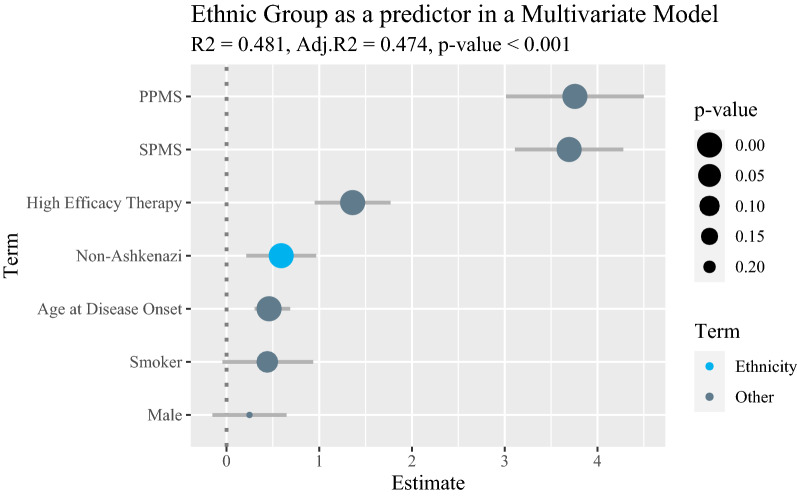
Ethnic group as a predictor in a multivariate model, this figure plots the estimates of the terms included in a multivariate regression model predicting MSSS (Multiple Sclerosis Severity Score). The terms plotted are organized by estimate value and are expressed in circles sized reversely to their p value, as depicted in the legend titles "p-value". Ethnicity is marked in the plot by a different color (blue) for emphasis. Ethnicity is shown to be a significant predictor of MSSS with an estimate value larger than those of Smoking, Date of onset, and Male sex.

In model 1, Non-AJ origin was a significant predictor of MSSS with a 0.590 increase in MSSS compared to Ashkenazi patients (CI 0.214, 0.967, *p* = 0.002, supplementary table 3 and Fig. 1). Disease subtype and High efficacy treatment were stronger predictors, while other factors such as age at onset, sex and smoking were significant with a lower impact (beta value), or non-significant.

When comparing the models using ANOVA, it was found that model 1 (including ethnicity) had significantly less variation (*p* < 0.001, supplementary table 4), and R-squared was higher by 0.01.

## Discussion

We found that Non-AJ MS patients differed from their Ashkenazi counterparts in several demographic characteristics. The ratio between females and males in the AJ group was 2.7:1, similar to reports in European countries^30,31^, while Non-AJ patients had a ratio of 1.6:1, smaller than that of any ethnic population recently studied^32^. Non-Ashkenazi patients in our sample were younger at the time of EDSS evaluation and at disease onset compared to Ashkenazi patients, these differences in age remained consistent when analyzing Israeli natives separately and thus cannot be attributed to immigration effect. The findings regarding younger age amongst Non-Ashkenazi patients can be compared to reports of earlier onset in Hispanic Americans and African Americans compared with Caucasian Americans^10,33^.

Non-AJ patients demonstrated a higher MSSS compared to AJ when controlling for age at EDSS and age at onset, both of which were found to differ between the groups and are potentially significant confounders affecting disability. MSSS was significantly higher in Non-AJ patients among those with RRMS and in females. This comparison did not reach significance in males, possibly due to smaller sample sizes limiting analysis. When focusing on male patients with RRMS we observed that Non-AJ patients were often treated more aggressively with more efficacious agents upon disease onset. This result might serve as evidence for a more severe disease phenotype at presentation in Non-AJ patients and might also mask differences in progression rate that otherwise would have been expressed by distinct MSSS results.

To control for the effect of immigration on disease severity^34^, we separately examined phenotype for immigrants and Israeli natives. Israeli natives showed the same effect of ethnicity on disease progression rate as described in the general sample, with a higher mean MSSS in native Non-AJ patients compared to native AJ patients.

Finally, in a multivariate regression model predicting MSSS, adjusted to disease subtype and high-efficacy treatment, Non-AJ ethnicity was the most notable epidemiological predictor even among factors previously known to considerably affect disease phenotype such as age at disease onset^35^, sex^27^ and smoking^28^. Treatment with high efficacy drugs was associated with a higher MSSS. This result might reflect the reasoning behind the choice of treatment according to the presentation and prognostic factors of the patient, rather than the effect of the treatment itself on disease course.

Due to variety in methods used to analyze MSSS results in similar studies, it is difficult to compare the severity of phenotype in patients with Non-AJ ancestry to other ethnicities worldwide. In the linear regression model included in this study a mean MSSS difference of 0.590 was found between Non-AJ and AJ patients. In a study by Berg Hansen et al. differences in disease severity between non-western immigrants and Norwegian patients were demonstrated using a linear model and a mean MSSS difference between groups was found to be 2.17^16^. However, in this study it is not possible to elucidate whether immigration to Norway or Non-Western ethnicity was the key contributor to the findings. Additionally, in our study, the difference between groups in the mean MSSS when adjusted to age at EDSS using ANCOVA was 0.72 (higher in Non-AJ), while in a study by Seyman et al., which used age and sex matching, immigrants of Middle Eastern and North African ancestry had a higher mean MSSS by 0.45 compared to patients of European ancestry in Canada^18^. Importantly, an advantage of this Israeli cohort is derived from the unique National Health Insurance Law in Israel allowing similar accessibility to healthcare services regardless of socioeconomic background and therefore reducing this plausible bias.

Several limitations should be acknowledged including: the retrospective nature of the study, information bias regarding ethnic affiliation recorded according to patients report and inter-rater variability in regards to EDSS scores determined by different clinician.

In this study, we describe differences in MS phenotype between AJ and Non-AJ in Israel, primarily a narrow sex gap, higher rate of disease progression and earlier disease onset in Non-AJ patients compared to AJ patients. These conclusions should be taken into account when evaluating prognosis and in clinical decision making. Further investigation will shed light on the genetic pathophysiological factors explaining the differences in disease phenotype across ethnic groups.

## Supplementary Information

Supplementary Tables.

## Data Availability

The datasets used and/or analyzed during the current study are available from the corresponding author on reasonable request.
