# Supplementary material for: Differences in MS clinical and epidemiological characteristics between Ashkenazi and non-Ashkenazi Jewish patients in Israel: a retrospective single center study
Source: Sci Rep. 2022 Mar 16;12:4555. doi: 10.1038/s41598-022-08565-7 (PMC8927451; doi:10.1038/s41598-022-08565-7)
Supplement: Supplementary file 1 — Supplementary Tables. [file 41598_2022_8565_MOESM1_ESM.pdf]

Supplementary data

Differences in MS clinical and epidemiological characteristics between Ashkenazi and Non-Ashkenazi Jewish patients in Israel – A retrospective single center study

Karni Arnon., Ben Noon Gil. *, Shiner Tamara., Vigiser Ifat., Kolb Hadar., Regev Keren.*

**These authors contributed equally to this work*

Supplementary table 1. Disease phenotype by sex in patients with RRMS

	Female			Male		
	Ash N=192	Non-Ashkenazi N=112	*p* value	Ash N=66	Non-Ashkenazi N=59	*p* value
Age (years); mean (sd) / median [1stQ, 3rdQ]	47.1 (14.7)	41.2 (13.9)	**0.001**	45.0 [36.0;55.5]	37.0 [31.5;49.0]	**0.012**
Age at onset; mean (sd) / median [1stQ, 3rdQ]	34.6 (11.6)	31.8 (11.8)	**0.045**	32.0 [26.2;37.0]	31.0 [23.0;38.0]	0.257
Disease Duration (years); median [1stQ, 3rdQ]	11.0 [5.00;17.0]	7.00 [3.00;13.0]	**0.005**	10.0 [4.00;16.0]	8.00 [3.50;13.0]	0.079
EDSS; median [1stQ, 3rdQ]	1.00 [0.00;2.00]	1.00 [0.00;3.00]	0.454	1.50 [1.00;2.88]	1.50 [0.00;3.00]	0.695
Global MSSS; mean (SD) / median [1stQ, 3rdQ]	1.76 (2.12)	2.40 (2.55)	**0.026** **0.011*** **0.008****	1.77 [0.53;3.62]	1.77 [0.53;4.14]	0.705 0.742*
PI; median [1stQ, 3rdQ]	0.10 [0.00;0.21]	0.13 [0.00;0.38]	0.236	0.16 [0.06;0.33]	0.19 [0.00;0.40]	0.729
EDSS 6.0 and above; n (%)	9 (4.69%)	11 (9.82%)	0.133	4 (6.06%)	6 (10.2%)	0.515
Spinal cord involvement in MRI; n (%)	104 (54.2%)	59 (52.7%)	0.895	35 (53.0%)	35 (59.3%)	0.598
Posterior Fossa involvement in MRI; n (%)	78 (40.6%)	46 (41.1%)	1.000	38 (57.6%)	29 (49.2%)	0.445
Treatment Strategy; n (%)			0.954			**0.026**
Untreated	21 (11.0%)	13 (11.6%)		6 (9.09%)	9 (15.3%)	
Platform therapy	120 (62.8%)	71 (63.4%)		40 (60.6%)	25 (42.4%)	
High efficacy	15 (7.85%)	10 (8.93%)		2 (3.03%)	10 (16.9%)	
Escalation therapy	35 (18.3%)	18 (16.1%)		18 (27.3%)	15 (25.4%)	
Oligoclonal bands in CSF; n (%)	71 (77.2%)	56 (81.2%)	0.676	27 (77.1%)	33 (84.6%)	0.602

Supplementary table 1. Disease phenotype by sex in patients with RRMS

	Female			Male		
	**Ash N=192**	**Non-Ashkenazi N=112**	***p* value**	**Ash N=66**	**Non-Ashkenazi N=59**	***p* value**

* Controlled for age at EDSS using ANCOVA

** Controlled for age at onset using ANCOVA

p values in bold denote statistical significance ($p<0.05$).

Normally distributed variables are compared using student's *t* test and are presented with mean (SD), while non-normal variables are compared using Mann Whitney U test and are presented with median [1st quartile, 3rd quartile].

Supplementary table 1. Disease phenotype by sex in patients with RRMS

Supplementary table 1 presents differences in disease phenotype between groups with stratification to sex. Variables that were normally distributed according to Shapiro test (*p* value < 0.05) are presented in the format of mean (sd) and compared using Student T-test. The MSSS in females was compared using student T-test as accepted for continuous scales in large samples. Non-normal variables are presented by median [1st qurntile, 3rd quartile], and compared using Mann-Whitney *U* test. Categorial variables are compared using Chi-squared test and presented with count (%). *p* values in bold denote statistical significance (p<0.05). *p* values marked by one asterisk (*) are controlled for age at EDSS, while those marked with 2 asterisks (**) are controlled for age at onset using ANCOVA.

Supplementary table 2. Disease phenotype by immigration status

	Born in Israel			Immigrant		
	**Ashkenazi N=149**	**Non-Ashkenazi N=178**	***p* value**	**Ashkenazi N=181**	**Non-Ashkenazi N=29**	***p* value**
Age (years); mean (sd) / median [1stQ, 3rdQ]	49.2 (15.0)	41.5 (13.4)	**<0.001**	48.0 [39.0;59.0]	56.0 [37.0;64.0]	0.371
Female; *n* (%)	109 (73.2%)	108 (60.7%)	**0.024**	133 (73.5%)	21 (72.4%)	1.000
Age at onset; median [1stQ, 3rdQ]	35.0 (11.9)	31.8 (11.4)	**0.015**	35.0 [28.0;44.0]	37.0 [25.0;47.0]	0.614
Disease Duration (years); median [1stQ, 3rdQ]	12.0 [5.00;20.0]	8.00 [3.00;14.0]	**<0.001**	11.0 [5.00;20.0]	11.0 [7.00;20.0]	0.408
EDSS; median [1stQ, 3rdQ]	1.50 [1.00;4.00]	1.50 [0.00;4.00]	0.922	2.00 [1.00;4.00]	3.00 [1.00;6.00]	0.224
Global MSSS; mean (sd) / median [1stQ, 3rdQ]	2.76 (2.84)	3.25 (3.03)	0.136<br>**0.009***<br>**0.034****	2.13 [0.53;5.02]	3.54 [0.94;6.32]	0.408<br>0.338***
PI; median [1stQ, 3rdQ]	0.14 [0.02;0.36]	0.21 [0.00;0.50]	0.124	0.18 [0.06;0.36]	0.22 [0.10;0.40]	0.522
EDSS 6.0 and above; n (%)	29 (19.5%)	34 (19.1%)	1.000	34 (18.8%)	9 (31.0%)	0.204
Spinal cord involvement in MRI; n (%)	82 (55.0%)	99 (55.6%)	1.000	106 (58.6%)	18 (62.1%)	0.878
Posterior Fossa involvement in MRI; n (%)	60 (40.3%)	86 (48.3%)	0.178	92 (50.8%)	9 (31.0%)	0.075
Treatment Strategy; n (%)			0.547			0.595
Untreated	17 (11.4%)	22 (12.4%)		14 (7.78%)	1 (3.45%)	
Platform therapy	66 (44.3%)	81 (45.5%)		108 (60.0%)	21 (72.4%)	
High efficacy	15 (10.1%)	25 (14.0%)		27 (15.0%)	2 (6.9%)	
Escalation therapy	51 (34.2%)	50 (28.1%)		31 (17.2%)	5 (17.2%)	
Oligoclonal bands in CSF; n (%)	48 (71.6%)	90 (81.1%)	0.202	70 (80.5%)	12 (80.0%)	1.000

* Controlled for age at EDSS using ANCOVA

** Controlled for age at onset using ANCOVA

*** Controlled for age at immigration using ANCOVA

p values in bold denote statistical significance (p<0.05).

Normally distributed variables are compared using student's *t* test and are presented with mean (SD), while non-normal variables are compared using Mann Whitney U test and are presented with median [1st quartile, 3rd quartile].

Supplementary table 2. Disease phenotype by immigration status

Supplementary table 2 presents differences in disease phenotype between groups in Israeli Natives and in immigrants. Variables that were normally distributed according to Shapiro test (p value < 0.05) are presented in the format of mean (sd) and compared using Student T-test. The MSSS in Israeli Natives was compared using student T-test as accepted for continuous scales in large samples. Non-normal variables are presented by median [1st quartile, 3rd quartile], and compared using Mann-Whitney U test. Categorial variables are compared using Chi-squared test and presented with count (%). p values in bold denote statistical significance (p<0.05). p values marked by one asterisk (*) are controlled for age at EDSS, while those marked with 2 asterisks (**) are controlled for age at onset using ANCOVA. Three asterisks (***) indicates p value controlled for age at immigration using ANCOVA.

Supplementary table 3. Multivariate regression model predicting MSSS

**Term**	**β (95% CI)**	***p* value**
(Intercept)	1.410 (1.118, 1.703)	**< 0.001**
Non-Ashkenazi	0.590 (0.214, 0.967)	**0.002**
SPMS	3.695 (3.110, 4.279)	**< 0.001**
PPMS	3.757 (3.015, 4.500)	**< 0.001**
Smoker	0.440 (-0.044, 0.935)	0.074
Male	0.248 (-0.152, 0.647)	0.224
Age at onset	0.495 (0.302, 0.687)	**< 0.001**
High efficacy therapy	1.360 (0.951, 1.769)	**< 0.001**

Dependent variable: MSSS
N = 535
F-statistic = 69.92 on 7 and 527 DF, *p* value < 0.001
R-squared = 0.4815, adjusted R-squared = 0.4746
Continuous predictors are mean-centered and scaled by 1 s.d.

Supplementary table 3 describes estimates and their *p* value in a multivariate regression model predicting MSSS. The model is based on 535 observations after 2 were dropped due to missing data in one of the variables. *p* values in bold denote statistical significance (p<0.05) and continuous predictors are mean-centered and scaled by 1 sd.

Supplementary table 4. Comparison of model fit

	R-squared	Adjusted R-squared	*p* value*
Model excluding ethnicity	0.472	0.466	
Model including ethnicity	0.481	0.474	
Difference in R-squared	0.009	0.009	**>0.001**

*comparison done using ANOVA test
N = 535

Supplementary table 4. Hierarchical multiple regression analysis

Supplementary table 4 presents a comparison preformed using ANOVA to compare a multivariate model including ethnicity, MS subtype, sex, smoking, high efficacy therapy and age at onset, with a similar model

excluding ethnicity as a predictor. Both models included 535 observations, after dropping 2 observations due to missing data. *p* values in bold denote statistical significance ($p<0.05$).
